# Plasma Treatment of Metal Surfaces for Enhanced Bonding Strength of Metal–Polymer Hybrid Structures

**DOI:** 10.3390/polym17020165

**Published:** 2025-01-10

**Authors:** Dong Hyun Kim, Han Su Kim, Yunki Jung, Jin-Yong Hong, Young-Pyo Jeon, Jea Uk Lee

**Affiliations:** 1 Department of Advanced Materials Engineering for Information and Electronics, Kyung Hee University, Yongin 17104, Republic of Korea; spy04032@khu.ac.kr (D.H.K.); hansu@khu.ac.kr (H.S.K.); yunki0930@khu.ac.kr (Y.J.); 2 Hydrogen & C1 Gas Research Center, Korea Research Institute of Chemical Technology (KRICT), Daejeon 34114, Republic of Korea; jyhong@krict.re.kr; 3 Advanced Materials and Chemical Engineering, University of Science and Technology (UST), Daejeon 34113, Republic of Korea

**Keywords:** anodization, plasma treatment, 3D printing, metal–polymer hybrid, bonding strength

## Abstract

The adhesion between metals and polymers plays a pivotal role in numerous industrial applications, especially within the automotive and aerospace sectors, where there is a growing demand for materials that are both lightweight and durable. This study introduces an innovative technique to improve the adhesion between a metal and a polymer in hybrid structures through the synergistic use of anodization and plasma treatment. By forming a nanoporous oxide layer on aluminum surfaces, anodization enhances the interface for polymer binding. Plasma treatment further augments the surface properties by increasing the concentration of functional groups, thus allowing better polymer infiltration during the 3D printing process. Comprehensive analyses, including X-ray photoelectron spectroscopy, energy dispersive X-ray spectroscopy, and contact angle measurements confirm the substantial enhancement in the bonding strength achieved through this method. Additionally, cross-sectional analysis via focused ion-beam etching provides a detailed view of polymer integration into the treated layers. The findings suggest significant potential for these surface modification strategies to advance the development of lightweight, robust composites suitable for use in sectors such as automotive, aerospace, and consumer electronics.

## 1. Introduction

Adhesion between metals and polymers is critically important in various industrial applications, particularly in the automotive and aerospace sectors where the demand for lightweight yet durable materials is ever-increasing [1,2]. Achieving robust metal–polymer adhesion can significantly enhance product performance by reducing the weight and material costs while maintaining or improving the mechanical properties [3]. Among the various techniques to improve adhesion, metal anodizing has stood out as a highly effective method. This process not only prepares the metal surface by creating a nanoporous oxide layer, but it also facilitates stronger bonding with polymers, making it an indispensable technique in advanced manufacturing.

Metal anodizing is an electrochemical passivation process that enhances the natural oxide layer on metal surfaces, offering several advantages, making it a versatile process for different applications [4,5]. The anodizing process enhances corrosion resistance, improves surface hardness, and allows for better paint adhesion. Moreover, the creation of a controlled nanoporous structure on the metal surface provides an excellent substrate for polymer infiltration, which is crucial for improving the shear strength in metal–polymer joints. Various studies have demonstrated the effectiveness of anodized surfaces in applications ranging from electronics to construction, illustrating the process’s adaptability and efficacy in enhancing the properties of a material [6,7].

This process is applicable to a variety of metals, including aluminum, titanium, zinc, magnesium, niobium, and zirconium. Among these, aluminum alloys are the most widely used due to their extensive application in industries such as aerospace, architecture, electronics, and automotive engineering. Aluminum alloys, classified into series ranging from AL2xxx to AL8xxx [8,9,10], are modified with elements like zinc, magnesium, silicon, manganese, and copper to meet specific functional requirements. These modifications allow for the precise tailoring of the material properties for distinct industrial uses. In particular, AL6061 alloys demonstrate superior corrosion resistance, excellent formability, and a high strength-to-weight ratio [11,12].

Following metal anodizing, post-treatment processes are vital to further optimize the joint properties and ensure long-term performance. Techniques such as chemical etching and sealing can enhance the surface characteristics, thereby improving the durability and strength of the metal–polymer adhesion. In this context, injection molded direct joining (IMDJ) emerges as a key technology [13,14,15]. It enables the direct integration of metals and polymers without additional adhesives, streamlining the manufacturing process and reducing potential failure points. Understanding the role of post-anodizing treatments and optimizing IMDJ parameters are crucial steps in advancing the application of metal–polymer hybrid structures in industry.

**Figure 1:**
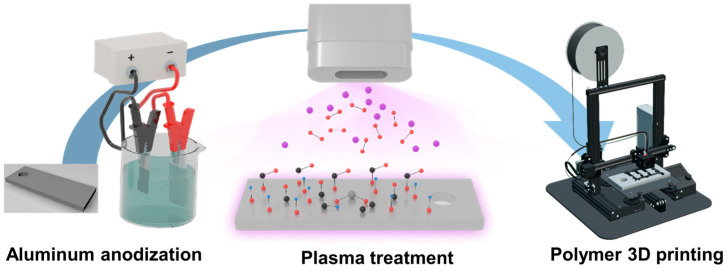
Manufacturing process of metal–polymer hybrids through the anodization and plasma treatment of aluminum substrates, followed by polymer 3D printing.

This study explored the enhancement of the bonding strength in metal–polymer hybrid structures through plasma treatment of metal surfaces and IMDJ processes. Utilizing anodization, a nanoporous oxide layer was formed on aluminum substrates, facilitating improved adhesion with polymers [16]. Plasma treatment further modified the surface, increasing the density of functional groups, which enhances the polymer infiltration during three-dimensional (3D) printing (Figure 1). The synergy of anodization and plasma treatment resulted in a significant increase in the bonding strength, as evidenced by X-ray photoelectron spectroscopy, energy dispersive X-ray spectroscopy, and contact angle measurements. Additionally, this study conducted an internal structural analysis of metal–polymer hybrids using cross-sectional images of the metal surface etched with a focused ion beam.

## 2. Experimental Methods

### 2.1. Materials

The aluminum alloy used in this study was AL6061-T6 (t3.0, Sungshin Jungmil, Incheon, Republic of Korea), composed of 97.6% aluminum, 0.8% magnesium, 0.7% iron, 0.4% silicon, and 0.4% copper, in accordance with the ASTM B209 standard [17]. The surface treatment of the aluminum alloy involved the sequential immersion in ethanol (98%, Samchun Chemical, Seoul, Republic of Korea), C-4000 (KG Chemical, Ulsan, Republic of Korea), NaOH (98%, Samchun Chemical, Seoul, Republic of Korea), and H_2_SO_4_ (95%, Samchun Chemical, Seoul, Republic of Korea). For the anodization process, a H_3_PO_4_ (85%, Samchun Chemical, Seoul, Republic of Korea) solution was used as the electrolyte. Platinum (Premion, 0.1 mm, 99.99%) served as the counter electrode, and the aluminum alloy functioned as the working electrode. The polymer filament material used for adhesion included polylactic acid (PLA, Cubicon, Seongnam, Republic of Korea).

### 2.2. Surface Treatment of Aluminum Alloys

The aluminum specimens first underwent sonication in ethanol for 30 min to remove surface impurities. Following sonication, the specimens were thoroughly rinsed with deionized (DI) water and sequentially immersed in C-4000, NaOH, and H_2_SO_4_ solutions for defined durations to perform surface pretreatment prior to anodization. After each immersion step, the specimens were rinsed with DI water before proceeding to the next stage. Once the pretreatment was complete, the rinsed specimens were dried in a convection oven at 80 °C for 6 h.

The pretreated aluminum specimens were then subjected to anodization in an H_3_PO_4_ solution. A constant current was applied using a power supply (EA-PS 2384-05 B, KMI system, Seongnam, Republic of Korea) set to 80 V and 0.1 A, with the pretreated aluminum acting as the working electrode and a platinum plate serving as the counter electrode. The anodization process was conducted for 15 min. After anodization, the specimens were rinsed with deionized (DI) water and dried in an oven at 80 °C for 6 h.

Surface activation via plasma treatment was performed using a plasma generation device (ArP Series, APP, Hwaseong, Republic of Korea), consisting of an RF power generator and a plasma head unit producing a plasma beam. The output power was adjusted to achieve various exposure times, ranging from 10 to 60 min. Six plasma-treated samples (P10 to P60) were prepared, with each sample corresponding to its respective treatment duration. The P10 sample, exposed to plasma for 10 min, was used as a reference in the subsequent analysis and discussion.

### 2.3. Preparation of Metal–Polymer Hybrid Structures

Upon completion of the surface treatment of the aluminum samples, the polymer injection was precisely applied onto the treated surfaces using a 3D printer (Core 200, Making Tool, New Delhi, India). This process resulted in the formation of a structurally robust single-lap-joint configuration, characterized by a significant bonding area measuring 5.0 × 10.0 mm. For further details regarding the metal–polymer hybrid fabrication process and the shear strength measurements, refer to Table 1 which provides comprehensive information categorized by factors and corresponding levels.

### 2.4. Bonding Strength Measurement

**Figure 2:**
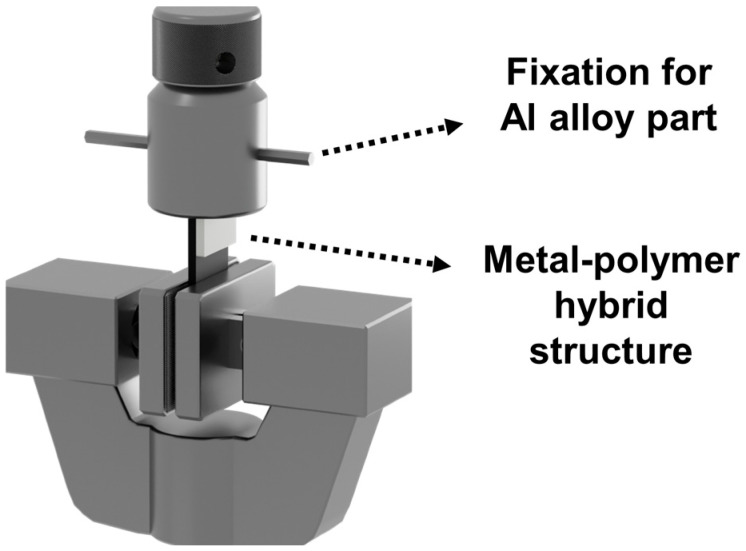
Measurement of bonding strength between the metal and polymer.

In accordance with the international standard ISO 19095-2:2015 [18], test specimens were fabricated following the overlapped specimen guidelines, specifically designed as type B. The bonding strength test was subsequently performed, as illustrated in Figure 2. Using a precision shear testing machine (AGS-X series, Shimadzu, Kyoto, Japan), the shear strength measurement was conducted at a controlled rate of 5 MPa/min. A total of seven samples were prepared, including both anodized and plasma-treated variations. Each sample underwent a comprehensive shear strength test, with ten repetitions performed for each. To ensure accuracy, the final shear strength values were calculated as the average of the eight test results, with the highest and lowest values excluded.

### 2.5. Characterization

A field emission scanning electron microscope (FE-SEM, LEO SUPRA 55; GENESIS 2000, Carl Zeiss, EDAX, Jena, Germany) was used to examine the surface morphology before and after anodization and plasma surface treatment. Additionally, energy dispersive X-ray (EDX) mapping integrated with an FE-SEM was employed to analyze the surface composition. For the SEM analysis, we used an accelerating voltage of 10.00 kV thermal field emission type electron gun and followed the standard sample preparation techniques to ensure the accuracy of the surface morphology analysis. The hydrophilicity of the surface, resulting from the anodization and plasma treatment, was evaluated by measuring the water contact angle (WCA). For each aluminum sample subjected to anodization and plasma treatment, WCA measurements were performed by dispensing 1.5 µL of water, using a contact angle measurement system (Phoenix 300, SEO Co., Ltd., Suwon, Republic of Korea). Furthermore, changes in the surface composition of the aluminum, induced by the anodization and plasma treatment, were analyzed using X-ray photoelectron spectroscopy (XPS, K-Alpha, Thermo Electron, Warriewood, Australia). During XPS analysis, a Kα radiation source with a power of 2000 eV was used for generating the photoelectrons. The survey results were obtained in the CAE (constant energy) mode using two different pass energies and energy steps. For the wide peak scan, the pass energy was set at 200 eV and the energy step size at 1 eV. For the narrow peak scan, a pass energy of 50 eV and an energy step size of 0.1 eV were applied. The aluminum (Al) samples were classified into three groups: bare Al, anodized Al, and Al treated with plasma for 10 min (P10), to assess surface changes before and after the anodization and plasma treatment.

## 3. Results and Discussion

### 3.1. Surface Characterization of Anodized and Plasma-Treated Aluminum

**Figure 3:**
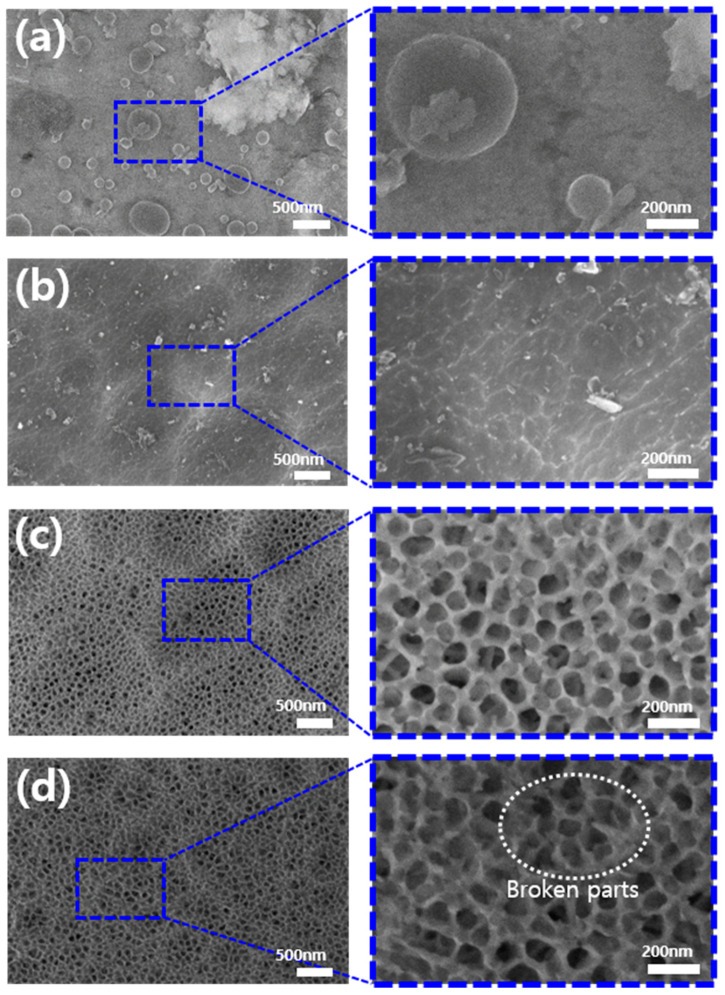
Surface morphology of (**a**) bare Al, (**b**) Al after pre-treatment, (**c**) anodized Al surface, and (**d**) plasma-treated Al with magnified images.

Figure 3 shows the surface morphology of untreated Al, pretreated Al, anodized Al, plasma-treated Al alloys as observed by the FE-SEM. Figure 3a reveals various impurities, including intermetallic particles of different sizes, which appeared as circular structures on the bare metal surface. Additionally, contaminants such as environmental residues and minerals were identified on the untreated Al alloy surface. These findings underscore the importance of the pretreatment and anodizing processes in modifying the surface characteristics of the AL 6061 alloy.

Figure 3b shows the surface morphology of the pretreated Al sample. Despite the presence of dust during analysis, it is clear that surface impurities, such as intermetallic particles and contaminants, were effectively removed. Additionally, shallow crater-like features were visible, caused by the etching effect of the basic pretreatment solution (NaOH) on the alloy surface. At higher magnification, a wavy boundary was observed on the surface, resulting from excessive etching with a basic solution, followed by exposure to an acidic solution, which caused the boundaries to blur. These changes demonstrate the effectiveness of the pretreatment process in removing impurities and reshaping the surface morphology.

Figure 3c illustrates the nano-porous surface structure resulting from the anodization of the pretreated aluminum sample. The cathodic oxidation process involves the reaction between Al^3+^ ions on the aluminum surface and O^2−^ ions from the electrolyte, leading to the formation of a dense Al_2_O_3_ oxide layer. As the oxide layer develops, electrical resistance increases, hindering the migration of both anions and cations, thereby suppressing further growth. The oxide film can be divided into an inner barrier layer and an outer anion-containing layer. During the oxide film’s growth, the electric field induces the formation of O^2−^ and OH^−^ oxygen bubbles within the outer layer of the film. The porous structure depicted in Figure 3c results from the escape of these oxygen bubbles from the oxide film, leading to the development of a well-defined nano-porous surface. This process demonstrates the formation of a controlled nano-porous structure via anodization, highlighting the intentional modification of surface characteristics. The observed nano-porous structure confirms the successful and precise execution of the anodization process, demonstrating its effectiveness in engineering specific surface features for various applications.

Figure 3d presents the image corresponding to sample P10, which was exposed to plasma treatment for 10 min, after the anodization process. No significant changes in structural integrity or pore size were observed following the anodization process. However, closer inspection reveals that some branches experienced disruption and breakage due to the intense ion bombardment during plasma exposure. The broken areas observed in Figure 3d suggest that plasma surface treatment can induce physical damage to the nano-porous structure formed by anodization, though such damage does not result in substantial alterations to the overall structure.

In previously reported studies on the anodic oxidation of the aluminum alloy, the “honeycomb”-like ordered porous layer expanded while the cell walls thinned, as the anodizing time increased. Eventually, some nanopores merged into larger pits, forming a “bird’s nest”-like structure [19]. Furthermore, more aggressive anodizing conditions, such as the use of phosphoric acid and elevated temperatures, resulted in extensive dissolution of the nanopore walls, leading to the collapse of the top part of the oxide during anodizing. This process produced a “bird’s nest”-like structure atop the honeycomb, characterized by microscale pits containing nanopores, which facilitated the penetration of 3D printed polymer into the aluminum surface pores [20]. In our experiments, although the plasma treatment was not performed simultaneously with anodization, its application to the anodized aluminum surface led to the collapse of pore walls, resulting in micro-pits resembling the “bird’s nest” structure described in the literature. While the chemical changes of the Al surface induced by the plasma treatment will be discussed later, the formation of micropits due to structural collapse may promote polymer penetration during the 3D printing process, potentially improving the strength of metal–polymer adhesion.

**Figure 4:**
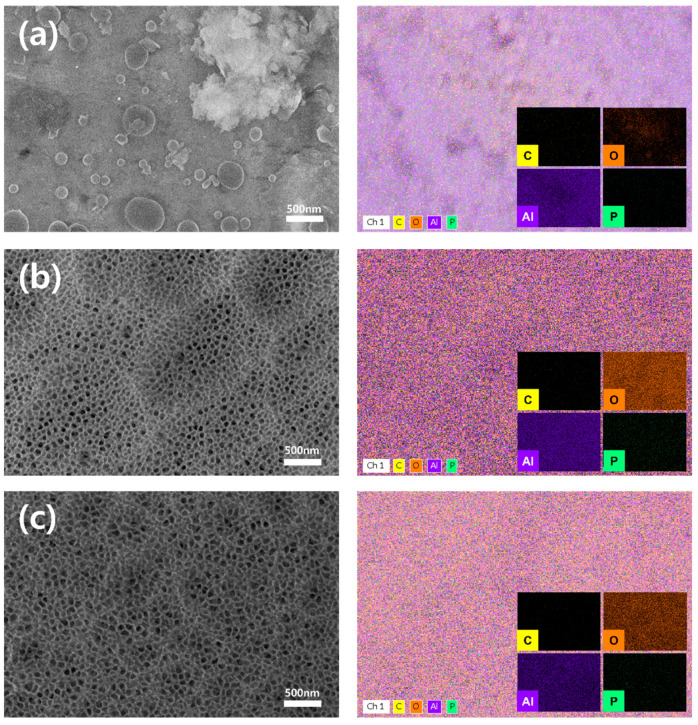
EDX mapping of (**a**) bare, (**b**) anodized, and (**c**) plasma-treated Al surfaces for four types of the elements: carbon (C), oxygen (O), aluminum (Al), and phosphor (P).

The FE-SEM images in Figure 4a–c provide EDX analysis results showing the elemental composition of three distinct samples: untreated Al, anodized Al, and fully processed samples subjected to both anodization and plasma treatment. Figure 4a presents the EDX mapping of bare Al, where prominent purple markings represent aluminum elements, with only trace amounts of other elements detected. Notably, anodization and plasma treatment result in a significant increase in the oxygen content, represented by orange markings on the surface (Figure 4b,c).

The elemental composition, presented as atomic percentages and determined through EDX mapping analysis, is summarized in Table 2. In the untreated Al sample, the elements detected and their respective atomic percentages were as follows: Al (76.97%), O (11.34%), C (11.69%), and P (0%). After anodization, the atomic percentages of Al and C decreased significantly, from 76.97% to 45.1% for Al and from 11.69% to 3.14% for C, respectively. In contrast, the oxygen content (O) increased substantially, from 11.34% to 51%. The trace presence of phosphorus (P), used as part of the anodizing electrolyte, was measured at 0.75%. Samples subjected to plasma surface treatment displayed similar trends to those of the anodized sample, with a reduction in the Al content and a slight increase in the O and C contents.

EDX analysis confirmed that the proportions of Al and C decreased, while the proportion of O increased significantly, as an oxide layer formed on the Al surface during the anodic oxidation process and plasma treatment. To bond heterogeneous polymers to a porous metal surface, the size and structure of the pores, along with the surface elemental composition, play a critical role [21,22,23,24,25]. The aluminum surface, rich in hydroxyl groups, can chemically interact with the polymer, enhancing the bonding strength. The proportion of hydroxyl groups on the aluminum surface is higher when phosphoric acid is used as the electrolyte compared to other electrolytes, such as sulfuric acid [26]. Therefore, in addition to the physical bonding between the metal and polymer, the Al sample, with a high concentration of oxygen on its surface, can achieve closer contact with the polymer. The enhanced contact facilitates more efficient thermal energy transfer between the heterojunctions, minimizing energy loss.

**Figure 5:**
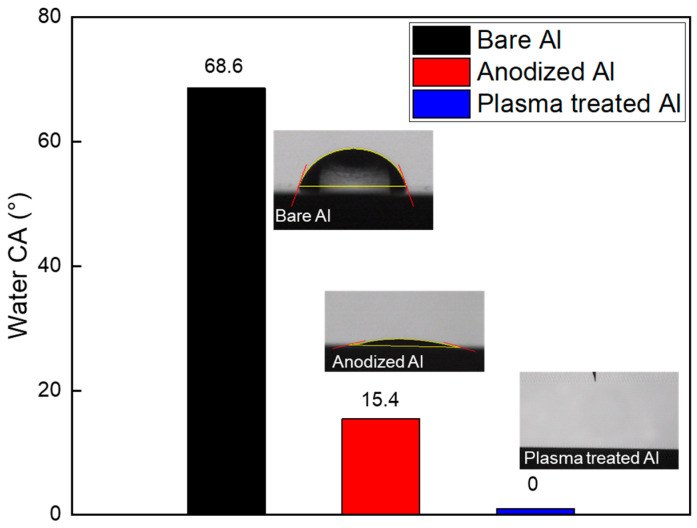
Water contact angle comparison for bare, anodized, and plasma treated Al.

Figure 5 illustrates the contact angle measurements and provides a comparison to validate the hydrophilicity of the surface following various aluminum surface treatments. The untreated Al initially exhibited an average contact angle of 68.6°, indicating a relatively hydrophobic surface. However, after electrochemical treatment, the contact angle of the anodized Al decreased markedly to 15.4°, signifying a transition to a hydrophilic surface. Further anodization, followed by plasma surface treatment, reduced the contact angle to nearly 0° due to the introduction of oxygen-rich functional groups on the surface. This outcome suggests the formation of a superhydrophilic surface, which exceeds conventional hydrophilic surface treatment [27]. Such a surface facilitates the complete spreading of contacting liquids and promotes the deeper penetration of injected polymer fluids.

**Figure 6:**
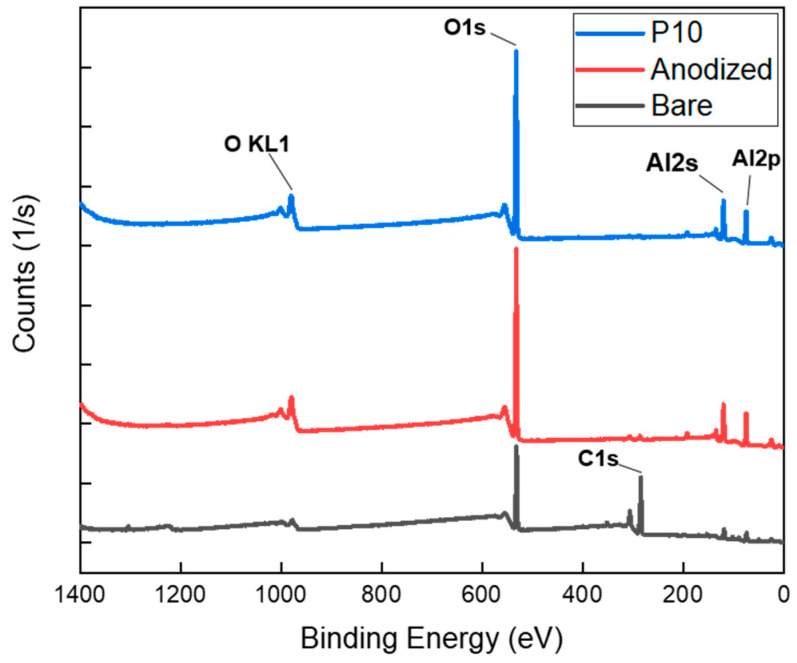
Comparison of XPS wide peak analysis: bare, anodized, and plasma treated (P10) Al surface.

Figure 6 presents the XPS analysis, comparing untreated Al, anodized Al, and Al samples subjected to subsequent plasma treatment, all showing wide scan peaks. Anodization notably induced a sharp increase in the O1s content on the surface, accompanied by a substantial reduction in the C1s peak compared to those of the untreated Al. However, a comparison of the XPS spectra before and after plasma treatment revealed no significant differences in the broad peak. These results suggest that while anodization substantially modified the surface’s elemental composition, plasma treatment did not have a pronounced effect on the elemental composition of the Al surface. This observation is consistent with the results of the EDX elemental analysis conducted through FE-SEM.

**Figure 7:**
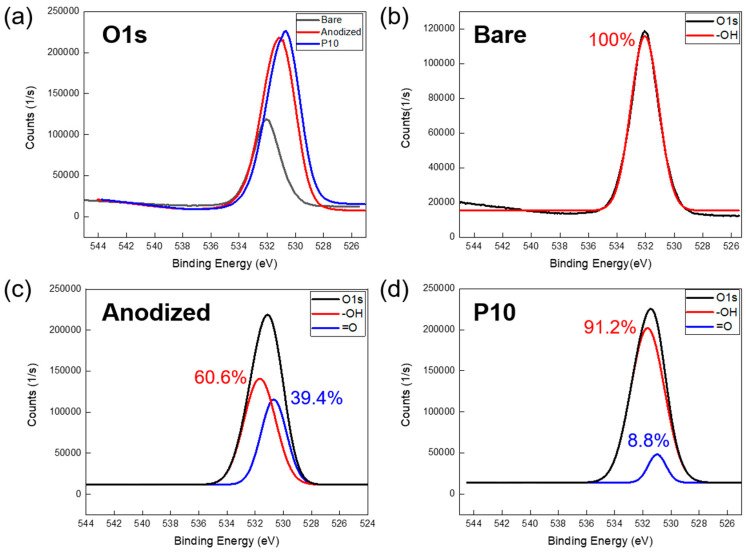
XPS analysis: (**a**) O1s narrow peak comparison for three samples. Graph of O1s deconvolution peaks: (**b**) bare Al, (**c**) anodized Al, and (**d**) plasma treated Al.

The XPS analysis, focusing on the narrow and deconvoluted peaks in Figure 7, more clearly elucidates the differences in peak characteristics compared to the wide-peak analysis, observed before and after the surface treatments. Figure 7a, which presents a magnified view of the O1s peak, shows an increase in peak intensity and a shift towards lower binding energy as the surface treatment progresses. Figure 7b–d provides a detailed analysis of the O1s deconvoluted peaks for each sample, which is further supported by the data in Table 3. Table 3 offers information on the energy levels and bonding states of each orbital, aiding in the interpretation of the chemical bonding characteristics on the Al surface.

Figure 7b presents the deconvoluted O1s peak of the bare aluminum surface, revealing the presence of only hydroxyl (–OH) groups on the surface, with no evidence of additional surface structures. Figure 7c,d illustrates the deconvoluted O1s peaks of the anodized and plasma-treated Al surfaces, respectively. In Figure 7c, an XPS analysis of the Al surface after anodization, clarifies a rightward shift of the entire O1s peak along with changes in its deconvoluted components. Notably, 39.4% of oxygen double bonds (=O) have appeared, while the proportion of the –OH functional group at 531.9 eV has decreased from 100% to 60.6%. Figure 7d highlights the impact of plasma treatment, showing a significant increase in the –OH functional groups in the O1s spectrum. The content of the –OH groups rise from 60.6% to 91.2%, indicating plasma-induced surface activation, characterized by a substantial increase in the surface energy. These detailed XPS findings underscore the transformative effects of anodization and plasma treatment on the Al surface chemistry, highlighting shifts in the functional groups and surface energy values. These changes are critical for understanding material behavior and optimizing potential applications.

**Figure 8:**
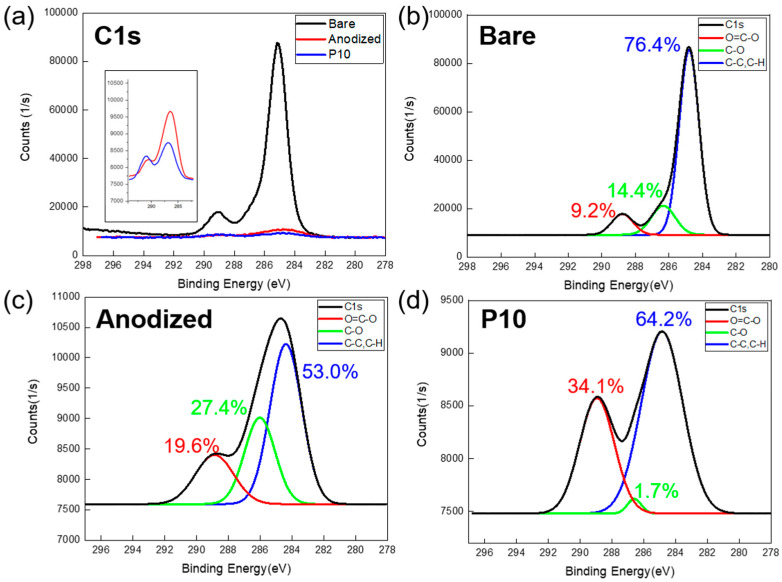
XPS analysis: (**a**) C1s narrow peak comparison for three samples. Graph of C1s deconvolution peaks: (**b**) bare Al, (**c**) anodized Al, and (**d**) plasma treated Al.

Figure 8 shows the C1s orbital peaks for all three samples, along with the corresponding deconvolution graphs for each sample. Table 4 offers C1s orbital information on the energy levels and bonding states of each orbital. Figure 8a reveals a clear trend across the three sample types, indicating a reduction in the presence of hydrophobic carbon on the surface, attributed to both the anodization and plasma surface treatment. Figure 8b depicts the C1s deconvolution peak for the bare aluminum sample. The untreated aluminum surface lacks a porous anodized layer and contains a minimal amount of carboxyl groups (O=C–O), a characteristic functional group in the C1s spectrum. Figure 8c demonstrates a synergistic effect between the nanoporous layer and the increased content of carboxyl groups following anodization.

Compared to the pre-plasma treatment state, there was a significant increase in the carboxyl group content after exposure to the plasma beam. Figure 8d demonstrates an increase in the percentage of carboxyl groups from 19.6% to 34.1% following plasma treatment, as evidenced by a pronounced rise in the shoulder peak. These observed changes in the C1s peaks highlight the significant impact of anodization and plasma treatment on surface chemistry, particularly in increasing the presence of oxygen-containing functional groups. This transition is crucial for improving surface wettability (water contact angle of 15.4° for the anodized Al and nearly 0° for the plasma-treated Al, Figure 5) and enhancing the material’s suitability for subsequent applications.

### 3.2. Bonding Strength Measurements of Metal–Polymer Hybrids

**Figure 9:**
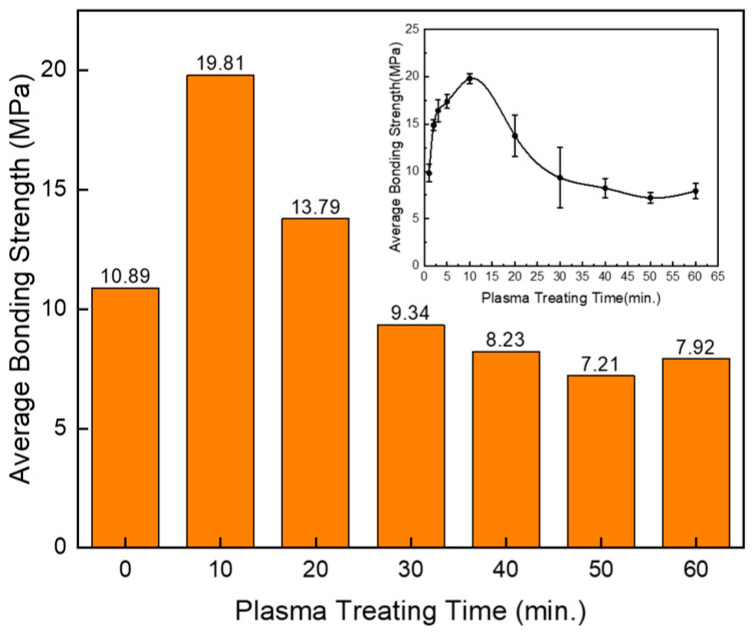
Comparison of average bonding strengths of PLA–Al heterojunction samples, as a function of plasma surface treatment duration. The inset image displays bonding strength measurements taken at 2.5 min intervals to verify the effects of plasma treatment before reaching the 10 min mark.

Surface-treated aluminum–polymer hybrid samples with a contact area of 5 × 10 mm^2^ were fabricated using 3D printing, in accordance with ISO 19095-2:2015 international standards. Figure 9 presents a bar graph illustrating the bonding strength values of PLA–Al hybrid samples, with the Al subjected to plasma surface treatment for durations exceeding 10 min. The plasma treatment was conducted in 10 min increments, up to a total of 60 min. Notably, the bonding strength increased significantly after 10 min of plasma treatment, reaching 19.81 MPa, nearly doubling the strength of the sample without plasma treatment, which was 10.89 MPa. Observing the upward trend in the bonding strength, a notable 15 MPa was recorded after just 2.5 min of plasma treatment, with the strength gradually increasing to reach its peak value after 10 min of treatment. Introducing surface activation by increasing the polarity for a porous structure on the Al surface through plasma treatment resulted in a significant improvement in the bonding strength. This chemical modification enhanced the penetration capability of the injected polymer fluid during the fabrication process.

**Figure 10:**
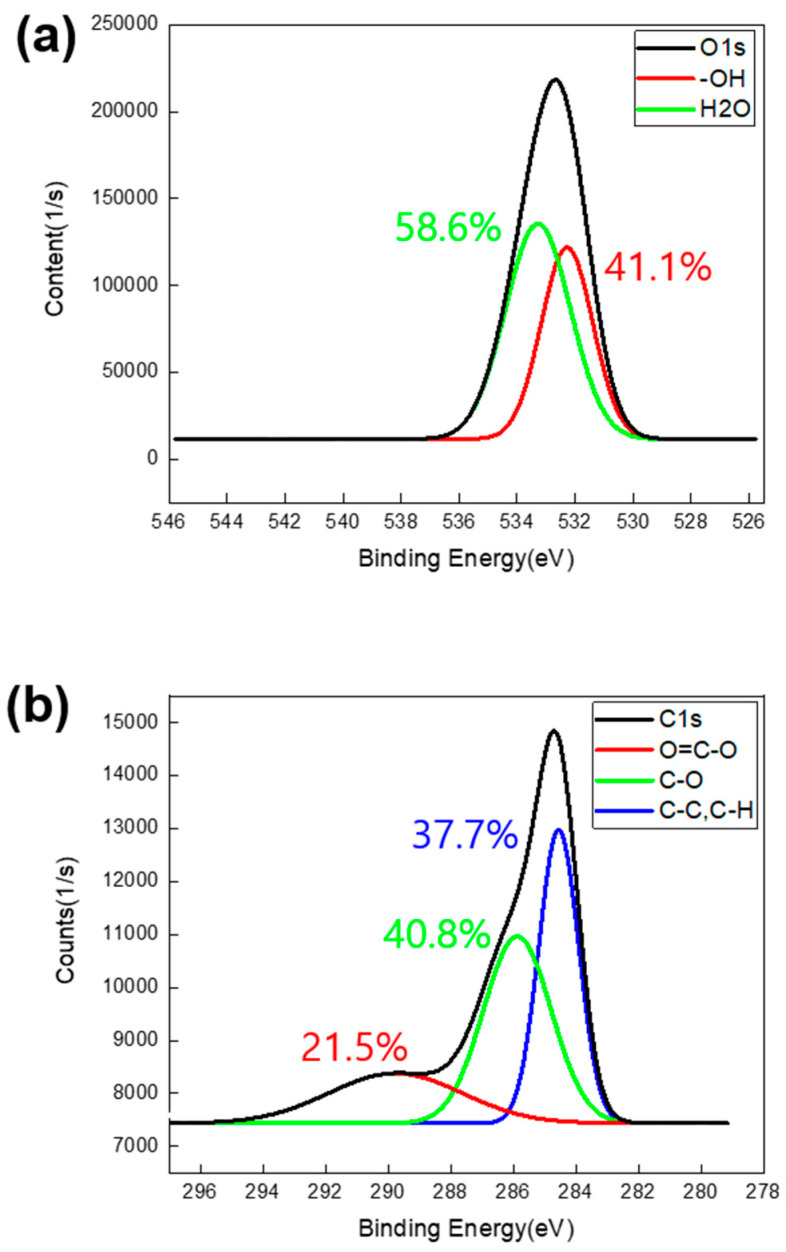
XPS deconvolution peaks of the PLA–Al heterojunction sample, exposed to plasma for 20 min: (**a**) O1s and (**b**) C1s orbits.

However, prolonged exposure to the plasma beam reduced the bonding strength. When the treatment duration exceeded 30 min, the bonding strength was measured to be lower than that of the untreated samples. The underlying cause of this behavior was elucidated through the FE-SEM and XPS analyses of the over-treated Al surface. Examination of Figure 10 shows that the O1s deconvolution peak of the chemically over-treated Al shifted to a higher binding energy (eV). The oxygen present in –OH and =O appeared in an H_2_O state, having already reacted with the small amount of –OH. Additionally, the content of the carboxyl group (O=C-O), which contributes to surface activation, decreased in the C1s deconvolution peak from 34.1% after 10 min of plasma treatment to 21.5% after 20 min of plasma treatment. These observations confirm the factors responsible for the decline in bonding strength observed in Figure 9 as the plasma beam exposure time increased.

### 3.3. Internal Structural Analysis of Metal–Polymer Hybrid Structures

**Figure 11:**
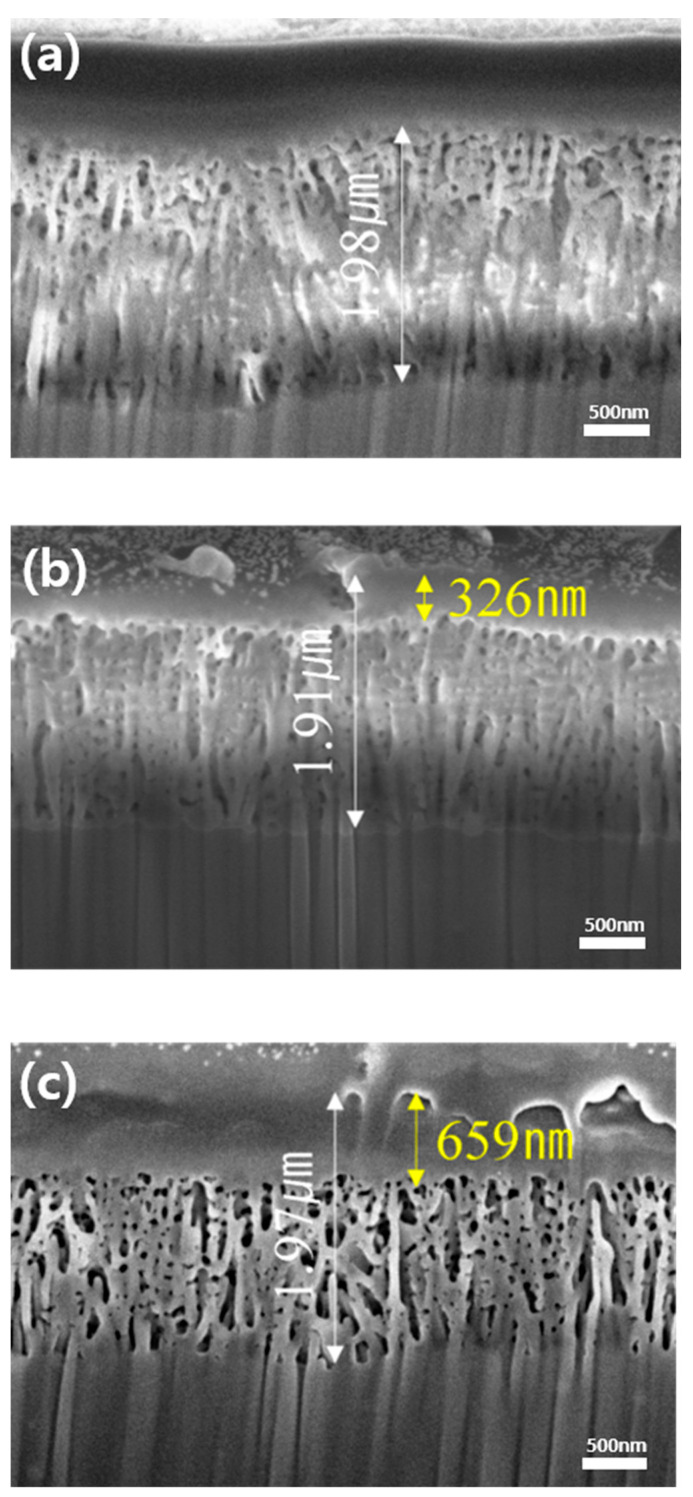
Cross-sectional FIB-SEM images showing (**a**) the anodized porous Al layer, polymer infiltrated (**b**) into the anodized porous layer, and (**c**) into the plasma-treated structure.

Figure 11 presents cross-sectional images of the Al surface etched with a focused ion beam (FIB-SEM). These images reveal the formation of a porous Al layer through anodization, as well as the infiltration of the PLA polymer into both the anodized and plasma-treated layers. The FIB-SEM image in Figure 11a shows the internal structure and depth of the anodized Al layer, highlighting distinct regions where polymer infiltration can occur. These permeable regions were formed by bubble escape during the H_3_PO_4_ anodization process. The anodized layer exhibits an average depth between 1.8 and 2.0 μm.

Figure 11b,c illustrates the internal structure of the anodized Al–PLA polymer hybrids, developed by 3D printing of PLA filament onto Al surfaces without and with plasma surface treatment. Notably, the infiltration depth of the PLA polymer increased significantly following plasma treatment, from approximately 326 nm to 659 nm, nearly doubling. This enhanced penetration can be attributed to a substantial increase in the surface functional group content [28,29,30], as confirmed by the XPS analysis after plasma treatment. The higher functional group density improves the polymer fluid’s mobility on the aluminum surface, facilitating deeper penetration into the anodized layer.

### 3.4. 3D Printing Applications of Metal–Polymer Hybrid Structures

**Figure 12:**
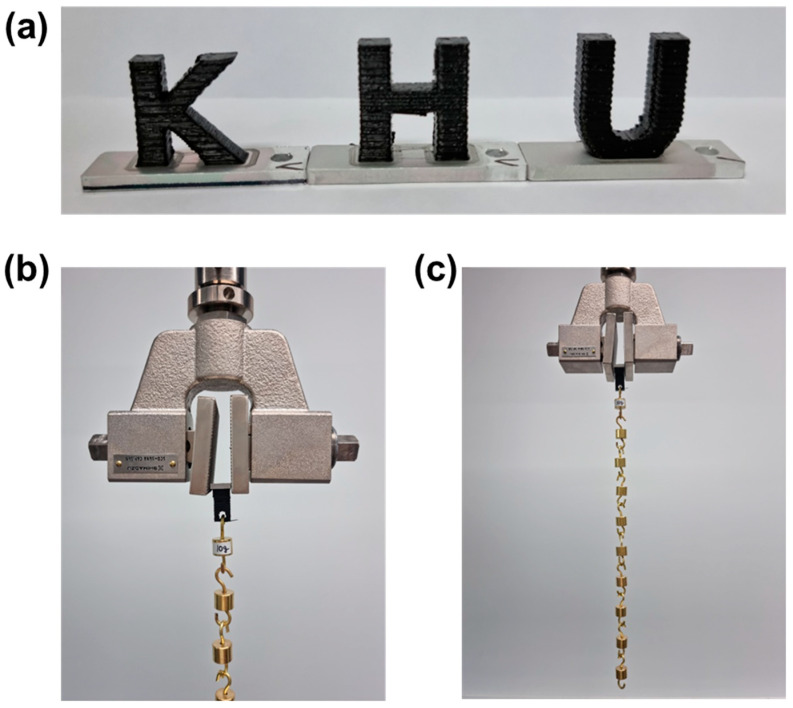
Photographs of 3D metal–polymer hybrids: (**a**) printed with Kyung Hee University’s logo (KHU), and (**b**,**c**) an enlarged view of the 3D printing area (25.0 × 10.0 mm), demonstrating stable support of ten 10 g weights, totaling 100 g.

Figure 12a presents an image of a PLA polymer that was successfully 3D printed onto an anodized Al surface. The complex text of Kyung Hee University’s logo (KHU) was printed with high precision and stability, facilitated by the nanopores created through anodic oxidation and the surface functional groups introduced via plasma post-treatment. This approach is anticipated to enable a wide range of metal–polymer hybrid applications across mobility, military, medical, and industrial sectors by integrating polymers with diverse structures, irrespective of shape and size, onto surface-treated Al substrates.

The adhesive properties of the 3D printed metal–polymer hybrids could be further improved through, e.g., plasma treatment on the anodized aluminum surface, well-control of the 3D printing conditions, and diversification of the 3D printed polymer resins [31,32]. As a simple example, Figure 12b,c shows photographs of a 3D printed metal–polymer hybrid sample with an enlarged 3D printing area (25.0 × 10.0 mm), which is stably supporting 10 weights of 10 g (total 100 g).

## 4. Conclusions

In conclusion, this study demonstrates the significant enhancement of bonding strength in metal–polymer hybrid structures achieved through the combined anodization and plasma treatment of Al surfaces. The anodization process increased the oxygen content on the Al surface from 11.34% to 51.02%, facilitating the formation of a nanoporous oxide layer that supports enhanced polymer infiltration. The subsequent plasma treatment further augmented the surface chemistry by increasing the content of hydroxyl groups from 60.6% to 91.2%, as evidenced by XPS analysis, resulting in a superhydrophilic surface with a contact angle approaching 0°. The bonding strength of Al–PLA hybrid structures improved significantly after 10 min of plasma treatment, reaching 19.81 MPa, nearly doubling the strength of the sample without plasma treatment, which was 10.89 MPa. However, an extended plasma treatment duration beyond 30 min resulted in decreased bonding strength, attributed to the reduction in carboxyl group content and the formation of micropits on the Al surface. These findings highlight the critical role of controlled surface modifications in optimizing the adhesion properties and mechanical performance of metal–polymer hybrids. This research provides valuable insights for the development of lightweight, durable materials applicable in the automotive, aerospace, and electronic industries.

## Figures and Tables

**Table 1:** Factors and levels applied during the processes of generating metal–polymer hybrid structures.

Factor		Level
3D Printing	Nozzle temperature (°C)	210
	Bed temperature (°C)	100
	Chamber temperature (°C)	60
	Printing speed (mm/min)	5000
Adhesion method/Area (m^2^)		Single lap joint/5.0–10.0
Measuring speed (MPa/min)		5

**Table 2:** The atomic percentage of Al, C, O, and P obtained from each Al sample in EDX mapping.

Element	Atomic Percentage (%)		
	Bare Al	Anodized Al	Plasma-Treated Al
Al	76.97	45.10	44.07
C	11.69	3.14	3.70
O	11.34	51.02	51.54
P	0	0.75	0.69

**Table 3:** Chemical bonding information and binding energy values of O1s orbitals.

Element	Specific Information		Binding Energy (eV)
	Orbital	Bond	
O	O(1s)		531.7
		O_1_(–OH)	531.9
		O_2_(=O)	530.8

**Table 4:** Chemical bonding information and binding energy values of C1s orbitals.

Element	Specific Information		Binding Energy (eV)
	Orbital	Bond	
C	C(1s)		288
		C_1_(C–C, C-H)	285
		C_2_(C–O)	286.5
		C_3_(O=C–O)	289

## Data Availability

Data are contained within the article.
